# Optimal complementary feeding practices of children aged 6–23 months in three agro-ecological rural districts of Jimma zones of southwest Ethiopia

**DOI:** 10.1017/jns.2023.26

**Published:** 2023-03-27

**Authors:** Meseret Tamrat Gebretsadik, Dessalegn Tamiru Adugna, Anteneh Dirar Aliyu, Tefera Belachew

**Affiliations:** 1 Department of Nutrition and dietetics, Jimma University, Jimma, Ethiopia; 2 Department of Population and Family Health, Jimma University, Jimma, Ethiopia

**Keywords:** Agro-ecological area, Children aged 6–23 months, Optimal complementary feeding, Southwest Ethiopia

## Abstract

Despite the critical importance of complementary feeding, large proportions of children in developing countries are sub-optimally fed during 6–23 months of age. In Ethiopia, even though the government has been rolling out infant and young child feeding (IYCF) guidelines, the proportion of mothers adhering to the recommended optimal practices and its associated factors have not been assessed in different agro-ecological areas. Hence, the present study aimed to determine optimal complementary feeding practices and associated factors in three agro-ecological rural districts (high, mid and lowland) of southwest Ethiopia. A community-based cross-sectional study was carried out among 845 mothers-index young children 6–23 months Jimma zone. Multistage sampling was employed to select the study participants. Structured and pretested questionnaires were used to collect data and entered into Epi Data V.1.4.4.0. The data were analysed using SPSS version 20. Binary and multivariable logistic regressions were used to identify factors associated with optimal child-feeding practices. The significance of the association was determined at *P* < 0⋅05. The overall proportion of optimal complementary feeding practice (OCFP) was 9⋅4 % at 95 % CI (7⋅19, 11⋅08). The timely initiation of complementary feeding, minimum meal frequency, minimum dietary diversity and minimum acceptable diet was 52⋅2, 64⋅1, 17⋅2 and 12⋅2 %. Multivariable logistic regression showed that being in the highland districts, having good maternal knowledge, and mothers having primary school education, having a family size of less than six were positively associated with optimal complementary feeding practices. The findings showed that OCFP was low, especially in the midland agro-ecological districts.

## Introduction

Pregnancy through to 2 years of life is the critical window of opportunity to prevent malnutrition among children. The widest part of the critical period was covered by complementary feeding^(1)^. Even though exclusive breast-feeding is ideal nutrition for optimal growth and development in the first 6 months, complementary food is mandatory to meet the need for energy and micronutrients for the child after 6 months to fill the gap. Hence, optimal complementary feeding practice (OCFP) during 6–23 months plays a crucial role in physical growth, cognitive development, intellectual ability and strengthening immunity^(2–4)^.

Sub-optimal complementary feeding practice is a vital factor contributing to inadequate nutrient intake among infants and young children^(1,5)^. Different researchers showed factors that influence OCFP are socioeconomic status, educational status, child age, maternal knowledge and family size^(4–8)^.

Globally, few children received nutritionally adequate, diversified, age-appropriate frequency and timely initiation of complementary food. In the world, evidence showed that 73 % of children aged 6–23 months received solid, semi-solid or soft foods at 6–8 months, 52 % of mothers fed their children with minimum meal frequency (MMF) and only 29 % of children have a diversified diet^(9)^. In Ethiopia, 55 % of children consumed a minimum number of times appropriate for their age. Although Ethiopia established infant and young child feeding (IYCF) practice guidelines^(10)^, only 14 % of the children received minimum dietary diversity (MDD), and 11 % of them received a minimum acceptable diet (MAD) in Ethiopia^(11)^.

The World Health Organization (WHO) developed a core indicator to assess optimal Infant and Young Children Feeding (OCFP) practices by integrating breast-feeding and complementary foods to improve the nutritional status of children less than 2 years. Introduction of complementary feeding from 6 to 8 months, MMF, MDD and the MAD recommended components for OCFP. The minimum frequency of meals is defined as the children 6–23 months of age who received solid, semi-solid or soft foods at least the minimum number of times or more during the previous day. A MAD is referred to composite indicator of both MDD along with MMF used to ensure optimal growth and development^(12,13)^. Dietary diversity is a proxy for adequate micronutrient density of foods and a potentially helpful indicator of quality complementary food. Higher dietary diversity was associated with increased nutrient intake or better child nutritional status in developing countries^(5)^.

The complementary feeding practice of mothers on the attainment of the diversified diet and frequency of meals appropriate to age contributes to improving the optimal child-feeding practice^(14)^. Some evidence in the Eastern part of Ethiopia and Kenya shows the variation of MMF and MDD in highland and midland agro-ecological areas^(6,15,16)^.

Therefore, it is crucial to understand different agro-ecological areas may affect the practice of optimal complementary feeding. So, besides a better understanding of the magnitude and associated factors of OCFP, this study could provide evidence to improve practice in diversified production in the different agro-ecological areas. Furthermore, it may help for National Nutrition Program to design new interventions.

A few studies were conducted on optimal complementary feeding practices across two agro-ecological districts (highland and midland) in some parts of Ethiopia. However, Jimma zone is one of the areas known for farming cereal, fruit and vegetables (FV), coffee and chat (*Catha edulis*) as the zone has high, mid and low agro-ecology. Despite that, there is no evidence in the zone and the Oromia region concerning optimal complementary feeding practices in different agro-ecological rural districts. In Ethiopia, previous studies have used one or two indicators, but the findings inconsistence to conclude optimum complementary feeding practice. Hence, we used four indicators to obtain better information on optimum complementary feeding practices. Therefore, the present study aimed to determine the optimal feeding practices and associated factors among children aged 6–23 months in three agro-ecological rural districts (high, mid and lowland) of Jimma zone, Ethiopia.

## Materials and methods

### Study setting, design and population

A community-based cross-sectional study was employed from February to March 2020 in three agro-ecological rural districts of the Jimma zone: Dedo (highland), Mana (lowland) and Kersa (midland) districts. Jimma zone is located 337 km southwest of Addis Ababa, the capital city of Ethiopia. The zones have thirteen districts having a total population of over 2⋅2 million with an agro-ecological setting of highlands predominantly growing fruit, tubers and vegetables, midlands growing cereals and legumes, and lowlands producing mainly coffee^(17)^. Based on the report of the respective districts’ health offices in 2018, the estimated number of children aged from 6 to 23 months was 1651 in Kersa, 688 in Mana and 914 in Dedo.

The source population was mothers or caregivers of children 6–23 months residing in three rural agro-ecology districts of the Jimma zone. Mothers/caregivers of children were willing to participate in the study and children who lived at least 6 months in the selected districts, with the exclusion of children who were unable to communicate and those who received supplementation for the treatment of malnutrition.

A single population proportion formula was used to calculate the sample size by considering a 95 % confidence level, the desired precision of 5 % and the proportion of children having timely initiation of complementary feeding (50⋅9 %) from the previous study^(15)^ yielding 384, a design effect of 2 and 10 % non-response rate to make used make a final sample size of 845 mother–child pairs.

A multistage sampling technique was employed to select a representative sample from the study population. Initially, three districts were selected purposely based on their predominating agro-ecological characteristics in the Jimma zone. Then, in each selected district, seven kebeles from Dedo (highland), five kebeles from Mana district (lowland) and five kebeles from Kersa districts (midland) were included in the study by a simple random sampling method. For each kebeles, the sample size was allocated proportionally to the total number of children aged 6–23 months. Then, the study population was selected by using a systematic sampling method from each kebele by using the health extension worker's family folder. In households that had more than one eligible child, only the youngest child was selected. When children were not available in the selected house, they were excluded and replaced by the next nearest household. The process was continued until the next K.

### Variables

Dependent variables: optimal complementary feeding practice

Independent variables: Socio-demographic factors, maternal knowledge, IYCF practice, child morbidity and hygienic practice

### Data collection tool and measurement

Structured and pretested questionnaires were used to collect data on socio-demographic characteristics, dietary practice, maternal knowledge, health and related characteristics. Based on the WHO guidelines, dietary data were collected by using a 24-h recall method. Mothers/caretakers were asked to recall all food items given to their child in the past 24 h before the day of the survey. According to the WHO 2021 guidelines, MDD was computed by counting and summing the eight food groups: (1) grains, roots or tubers; (2) legumes and nuts; (3) vitamin A-rich FV; (4) other fruits or vegetables; (5) flesh foods (meat, poultry, fish, organ meat); (6) eggs; (7) dairy products (milk, yogurt, cheese) and (8) breast milk. MMF was determined based on the frequency of feeding per 24 h. A MAD was considered a composite indicator that relies on a combination of standards of MDD and minimum feeding from 24-h recall. Timely introduction of complementary foods considers as the proportion of infants 6–23 months of age who received solid, semi-solid or soft foods at 6 months. If the child fulfilled the above four indicators, the child is assigned as having OCFP^(12,13,18)^. Other questionnaires were constructed from different literature^(8,19,20)^. The knowledge index was created from nine questions. Each was coded as 1 if a participant's answer was correct or ‘0’ if the response was incorrect. Then, the knowledge score was calculated, and the cut-off was decided based on a mean score of 3⋅7 (±1⋅56). Mothers who responded correctly to four or more questions were considered good knowledge^(21)^. If the child had diarrhoea, cough and fever in the past 2 weeks preceding the survey, and the mother said ‘yes’ for one or more of these common morbidities, considered as the child has the illness.

### Data quality

The questionnaires were prepared in English and translated into Afan Oromo for data collection. A pre-test was done on 5 % of the questionnaires outside the study area 2 weeks before the collection of actual data. Ten trained nurse graduates who can fluently speak the local language participated in data collection. The data collectors and two supervisors met at the end of each day, discussed their performances and modified their issues to be corrected accordingly.

### Data analysis

The data were entered into Epi Data Manager and entry version 1.4.4.0 and exported to SPSS version 20.0 for analyses. Descriptive statistics were used to summarise the data, and chi-square (*χ*^2^) was used to show the association between food groups and the agro-ecological districts. It was used to see the crude association between each independent variable with the outcome variable was examined through bivariate logistic regression analysis. Then, all variables with a *P*-value less than 0⋅25 entered into a multivariable logistic regression to control the possible confounders and identify the predictors at a *P*-value of <0⋅05. Multicollinearity was checked by using a standard error of less than 2⋅0 and model fitness by the Hosmer–Lemeshow test (*P* = 0⋅781).

### Ethical clearance

The ethical clearance was obtained from the Institutional Review Board (IRB) of Jimma University, Institute of Health, Faculty of Public Health, with approval number (ref. HRPGC/536/2019). All mothers or caretakers involved in the study were informed about the purpose and told they could stop the interview at any time they wanted.

## Results

### Socio-demographic characteristics

Of 845 mother–child pairs, 843 (99⋅3 %) respondents gave complete responses. The mean age of children was 13 (±4⋅7) months and 44⋅5 % of participants were in 6–11 months. The majority of children were males (52 %). The mean age of mothers was 26⋅8 (±5), and the majority (54⋅2 %) of them were 25–30 years old. The majority of caretakers (92⋅5 %) were married. Nearly, all participants were Oromo (95⋅8 %) and Muslim (96⋅6 %). More than half of mothers/caregivers (63 %) and fathers (48⋅6 %) were unable to write and read, and almost all mothers were housewives (91⋅9 %). The average family size of study participants was six. One-third (33⋅1 %) of participants reported that their husbands exclusively decided on the household's economy (Table 1).

**Table 1.** Socio-demographic and health characteristics of the participants in Jimma zone, southwest Ethiopia, 2019

Variables	Frequency (*n*)	Percentage (%)
Age of child		
6–11	375	44⋅5
12–17	303	35⋅9
18–23	165	119⋅6
Sex		
Male	438	52
Female	405	48
Age of mothers (Years)		
<20	122	14⋅5
20–24	117	13⋅9
25–30	457	54⋅2
>30	147	17⋅4
Marital status		
Single	45	5⋅3
Married	780	92⋅5
Widowed	10	1⋅2
Separated	8	0⋅9
Ethnicity		
Oromo	808	95⋅8
Amhara	17	2⋅01
Tigre	12	1⋅49
Dawero	5	0⋅59
Kaffa	1	0⋅11
Religion		
Muslim	814	96⋅6
Orthodox	18	2⋅1
Protestant	10	1⋅2
Wakefata	1	0⋅1
Mother educational status		
Unable to write and read	531	63
Write read	59	7
Primary (1–8)	231	27⋅4
Secondary and above	22	2⋅6
Father educational status		
Unable to write and read	410	48⋅6
Write read	88	10⋅4
Primary (1–8)	264	31⋅3
Secondary and above	81	9⋅6
Maternal occupation		
Housewife	775	91⋅9
Farmer	23	2⋅7
Government employee	17	2
Private	25	3⋅3
Father occupation		
Farmer	739	87⋅7
Government employee	32	3⋅8
Private	65	7⋅7
Daily labourer	7	0⋅8
Household size		
1–3	145	17⋅2
4–6	306	36⋅3
>6	392	46⋅5
Decision-making power		
Mother and father jointly	222	26⋅3
Father	279	33⋅1
Mother	95	11⋅3
Family	135	16
Neighbours	112	13⋅3

### Health and related characteristics

More than half (52⋅4 %) of the mothers/caregivers delivered their last child at home. Regarding birth order, more than half of the children (63⋅7 %) were third and above children of their parents. Regarding comorbidity, (42⋅6 %) of children was exposed to illness in the last 2 weeks preceding the survey. More than one-third (36⋅8 %) of mothers provided an extra meal for their children during sickness (Table 2).

**Table 2.** Health and related characteristics of participants in Jimma zone, southwest Ethiopia

Variables	Frequency (*n*)	Percentage (%)
Delivery place		
Home	442	52⋅4
Health facilities	401	47⋅6
Birth order		
First	164	19⋅5
Second	142	16⋅8
Third and above	537	63⋅7
Morbidity report		
Yes	359	42⋅6
No	484	57⋅4
Type of morbidity (*N* 359)		
Diarrhoea	124	34⋅5
Cough	128	35⋅7
Intestinal parasites	92	25⋅6
Fever, tonsillitis	15	4⋅2
Feeding during illness (*N* 359)		
As usual	227	65⋅2
Additional meals	132	36⋅8

### Infant and young child feeding practices by agro-ecology

The mothers/caregivers (88⋅7 %) had practised breast-feeding, and 64⋅7 % continued breast-feeding until 12–23 months of the child's age. The overall prevalence of children who started complementary food at 6 months was 65⋅7 %. There is no statistical difference between agro-ecological production and timely initiation of complementary feeding at 6 months (*P* = 0⋅148). In the highland, the highest practice of MDD was observed (23⋅5 %). However, only 8⋅4 % of mothers fed their children based on the recommended MDD in the midland. One hundred forty-six (61⋅3 %), 128 (73⋅6 %) and 266 (61⋅7 %) mothers achieved the MMF among high, low and midland districts, respectively. The overall rate of MAD was 11⋅4 %, however, only 6⋅3 % of mothers met the MAD in midland. Regarding the OCFP, only 9⋅4 % of children met the optimum feeding practice, among these; a low proportion of children (4⋅4 %) received optimal child-feeding practice in the midland districts. There are statistically significant differences between the agro-ecological areas and the children ever breast-fed, continued breast-feeding and MMF (*P* < 0⋅005), MDD, MAD and OCFP (*P* < 0⋅0001) (Table 3).

**Table 3.** Child feeding practice across agro-ecology in Jimma zone, southwest Ethiopia

Variables	Overall *n* (%)	Highland *n* (%)	Lowland *n* (%)	Midland *n* (%)	*P*
Ever breast-feed					
Yes	748 (88⋅7)	204 (85⋅7)	164 (94⋅3)	380 (88⋅2)	<0⋅05*
No	95 (11⋅3)	34 (14⋅3)	10 (5⋅7)	51 (11⋅8)	
Continued BF					
Yes	545 (64⋅7)	132 (55⋅5)	118 (67⋅8)	295 (68⋅4)	<0⋅05*
No	298 (35⋅3)	106 (44⋅5)	56 (32⋅2)	136 (31⋅6)	
Introduction of Complementary Food (CF)					
Yes	440 (52⋅2)	150 (63)	87 (50⋅0)	203 (47⋅1)	0⋅148
No	403 (47⋅8)	88 (37)	87 (50⋅0)	228 (52⋅9)	
MDD					
Yes	128 (15⋅2)	56 (23⋅5)	36 (20⋅7)	36 (8⋅4)	<0⋅001**
No	715 (84⋅8)	182 (76⋅5)	138 (79⋅3)	395 (91⋅6)	
MMF					
Yes	540 (64⋅1)	146 (61⋅3)	128 (73⋅6)	266 (61⋅7)	<0⋅05*
No	303 (35⋅9)	92 (38⋅7)	46 (26⋅4)	165 (38⋅3)	
MAD					
Yes	96 (11⋅4)	41 (17⋅2)	28 (16⋅1)	27 (6⋅3)	<0⋅001**
No	747 (88⋅6)	197 (82⋅8)	146 (83⋅9)	404 (93⋅7)	
OCFP					
Yes	79 (9⋅4)	38 (16⋅0)	22 (12⋅6)	19 (4⋅4)	<0⋅001**
No	764 (90⋅6)	200 (84⋅0)	152 (87⋅4)	412 (95⋅6)	

BF, breast-feeding; MAD, minimum acceptable diet; MDD, minimum dietary diversity; MMF, minimum meal frequency; OCFP, optimal complementary feeding practice.

Continued BF, refers to children 12–23 months of age who were fed breast milk during the previous day.

Timely initiation refers to infants 6–23 months of age who received solid, semi-solid or soft foods at 6 months.

* Significant.

** Highly significant.

### Consumption of food items by children across agro-ecological districts, in Jimma zone, 2019

**Fig. 1.**
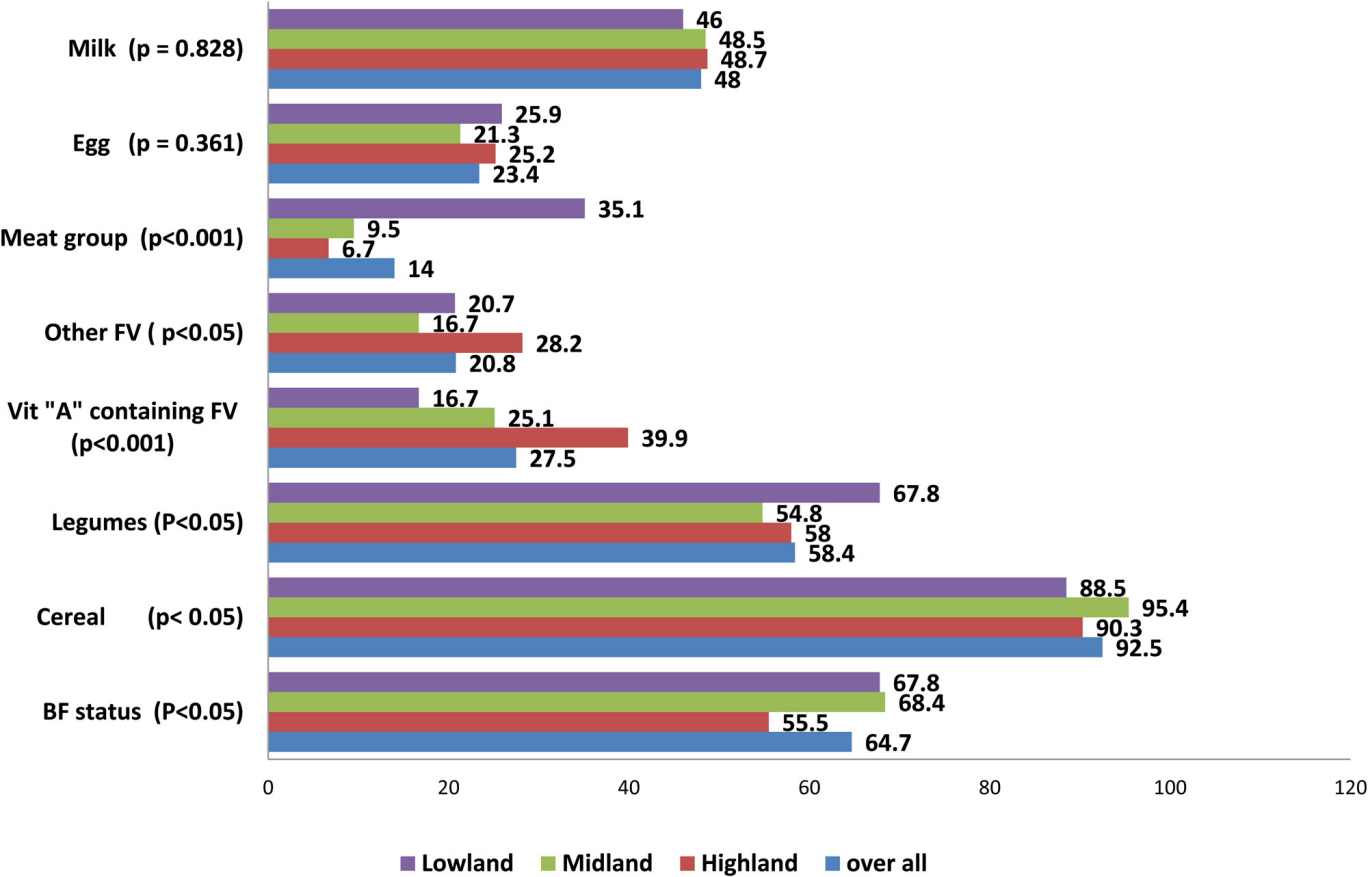
Types of food groups given to children aged 6–23 months across agro-ecological areas in Jimma zone, southwest Ethiopia. The solid blue bar represents the overall percentage of children who consumed the type of food groups; the red bar represents those children who ate the item of food groups in the highland; the green bar represents those children who consumed food groups in the midland and the violet bar represents children who ate food in the lowland. Chi-square (*χ*^2^) was used to show the association between food groups consumed by children and the three agro-ecological districts as **P < 0⋅0001 and * *P* < 0⋅005.

Overall, grain, root and tubers (92⋅5 %) were the most common food items consumed by children, and the least (14 %) were flesh foods in the 24 h preceding the survey. Cereals were the most commonly consumed in the highland (90⋅3 %), lowland (88⋅5 %) and midland (95⋅4 %) among three agro-ecological areas. The highest intake of legumes and nuts by children was (67⋅8 %) in lowland and highland (58⋅0 %). Flesh food consumption was higher in the lowland (35⋅1 %) (*P* < 0⋅001). The intake of vitamin A-containing FV and other FV among all participants was 27⋅5 and 20⋅8 %, respectively. However, the proportion of children who ate vitamin A-containing FV 39⋅9 % (*P* < 0⋅001) and other FV 28⋅2 % was higher in the highland (*P* < 0⋅05) There was no difference in consumption of eggs (*P* = 0⋅408) and dairy products (*P* = 0⋅828) (Fig. 1).

### Maternal knowledge of OCFP

Overall, 535 (42⋅7 %) mothers had good knowledge about complementary feeding practices. Of 535 mothers, the majority (72⋅4 %) were from the lowland, whereas the rest were from the high (37⋅4 %) and midland (33⋅6 %) (Table 4).

**Table 4.** Knowledge of mothers on IYCF in Jimma zone, southwest Ethiopia

Complementary feeding knowledge on	Had knowledge	Percent (%)
When to start CF feeding	535	63⋅5
Importance of CF	202	24 0
Consistency of CF	276	32⋅7
Local Household (HH) traditional food processing method	72	8⋅5
Frequency of feeding young children	493	58⋅5
Additional foods during illness	180	21⋅4
Risk of bottle feeding	488	57⋅9
How to prepare diversified complementary foods	185	21⋅9
Hand washing before food preparation	739	87⋅7
Knowledge score (good)		

Overall	Highland	Lowland	Midland	*χ*^2^	*P*
360 (42⋅7 %)	89 (37⋅4 %)	126 (72⋅4 %)	145 (33⋅6 %)	79⋅975	<0⋅001

### Factors associated with OCFP

On the bivariate regression analysis, agro-ecological area, maternal knowledge, child age, maternal education, father education and household size were significantly associated with OCFP. After controlling for the potential confounding agro-ecological area, maternal knowledge, maternal education and family size were significantly associated with OCFP. However, the age group and father's education were not associated with OCFP. Mothers/caretakers who lived in the highland area were 4⋅2 times more likely to give optimal complementary feeding to their children compared to mothers who lived in the midland agro-ecological zone [AOR = 4⋅215, 95 % CI (2⋅204, 8⋅062)]. Children from a household where the mother has good knowledge about OCFP were 3⋅95 times more likely to practice OCFP than those mothers who had poor knowledge [AOR = 3⋅952, 95 % CI (2⋅121, 7⋅366)]. Mothers who attended primary school and above were two times more likely to practice optimal complementary feeding [AOR = 2⋅817, 95 % CI (1⋅516, 5⋅235)] than mothers unable to read and write. Those households having 1–3 persons were 3⋅6 times [AOR = 3⋅649, 95 % CI (2⋅116, 6⋅292)] more likely to practice optimal complementary feeding compared with those households having greater than 4 and above persons (Table 5).

**Table 5.** Bivariate and multivariable logistic regression models predicting the likelihood of optimal complementary feeding practice among children aged 6–23 months in southwest Ethiopia

Variable	Complementary feeding practice		COR with 95 % CI	AOR with 95 % CI
	Appropriate	Inappropriate		
	*n* (%)	*n* (%)		
Agro-ecology				
Highland	38 (16)	200 (84⋅0)	4⋅12 (2⋅3161, 7⋅329)**	4⋅215 (2⋅204, 8⋅062)**
Lowland	22 (12⋅6)	152 (87⋅4)	3⋅139 (1⋅653, 5⋅96)**	1⋅738 (0⋅856, 3⋅529)
Midland	19 (4⋅4)	413 (95⋅6)	1	1
Maternal knowledge				
Good	62 (17⋅2)	298 (82⋅8)	5⋅703 (3⋅27, 9⋅944)**	3⋅952 (2⋅121, 7⋅366)**
Poor	17 (3⋅5)	466 (96⋅5)	1	1
Age group				
6–11	27 (7⋅2)	348 (92⋅8)	1	1
12–17	31 (10⋅2)	272 (89⋅8)	1⋅469 (0⋅856, 2⋅520)	1⋅06 (0⋅581, 1⋅943)
18–23	21 (12⋅7)	144 (87⋅3)	1⋅88 (1⋅029, 3⋅433)*	1⋅256 (0⋅631, 2⋅499)
Mother education				
Unable to read and write	26 (4⋅9)	505 (95⋅1)	1	1
Read and write	3 (5⋅1)	56 (94⋅9)	1⋅041 (0⋅305, 3⋅548)	0⋅392 (0⋅102, 1⋅511)
Primary and above	50 (19⋅8)	203 (80⋅2)	4⋅78 (2⋅898, 7⋅896)**	2⋅817 (1⋅516, 5⋅235)*
Father education				
Unable to read and write	25 (6⋅1)	385 (93⋅9)	1	1
Read and write	9 (10⋅2)	79 (89⋅8)	1⋅754 (0⋅789, 3⋅902)	1⋅526 (0⋅592, 3⋅932)
Primary and above	45 (13⋅0)	300 (87⋅0)	2⋅31 (1⋅385, 3⋅853)*	1⋅096 (0⋅586, 2⋅051)
Family size				
1–3	41 (28⋅3)	104 (71⋅7)	6⋅8 (4⋅206, 11⋅148)**	3⋅649 (2⋅116, 292)**
≥4	38 (54⋅4)	660 (94⋅6)	1	1

AOR, adjusted odd ratio; CI, confidence interval; COR, crude odd ratio.

* Significant at *P* < 0⋅05.

** Significant at *P* < 0⋅001.

## Discussion

The finding of the present study revealed that the prevalence of OCFP was 9⋅4 %. This finding is in agreement with the study finding from secondary data analysis of 2019 mini Ethiopia Demographic and Health Survey (EDHS) (9⋅7 %), Horo district (9⋅91 %) and Sidama (8⋅6 %)^(8,18,20)^. However, this study is much lower than the studies conducted in Ghana (15⋅7 %), Shasemene (30 %) and Amhara region (26⋅8 %)^(19,22,23)^. This discrepancy from previous Ethiopian studies might be the studies conducted in urban and adjacent to urban areas, mothers might have relatively better access to information about appropriate complementary feeding practices from different mass media compared with the present study of the urban area. The variation in finding from Ghana might be because the study was conducted during the post-harvest season. Moreover, in the present study, the practice of OCFP varied among agricultural diversification of foods. Children are at high risk of under nutrition in the first 2 years of life when there are limitations in the quality and quantity of their diets. Moreover, children aged 6–23 months are recurrently prone to infectious diseases like diarrhoea and upper respiratory infection due to common micronutrient deficiencies. Thus, optimum complementary feeding practice is needed to provide adequate energy and essential nutrients required for continued normal growth and development.

In the present study, 52⋅2 % of infants were introduced to solid, semi-solid or soft foods at 6 months, with almost similar findings across agro-ecological areas. The current figure is nearly in-line with the studies from Ethiopia (60⋅4–83⋅2 %)^(8,18,22,23)^, South Asia (57⋅4 %)^(24)^ and Indonesia (50 %)^(25)^.

Complementary foods for children should be varied and nutrient-dense in at least five food groups^(13)^. However, in the present study, only 15⋅2 % of children met the minimum requirement of five or more from the eight food groups, and the highest prevalence was in highland (26⋅9 %), lowland (23 %) and midland (9⋅5 %). The overall MDD is similar to the review of mini EDHS 2019^(18)^, studies in Horo^(8)^ and Sidama^(20)^ in Ethiopia. This finding is much lower than the review in southern Ethiopia from 6 to 9 surveys^(26)^ and Ghana^(19)^. In the case of southern Ethiopia, different projects are working on the awareness creation of child feeding practices, particularly on the inclusion of pulses and animal-source food in the diet of children. It might help mothers to look into legumes and animal products, hence it might increase the number of food groups consumed by children. The variation of the present finding from the Ghana study could be the study period. Thus, seasonality may have contributed to increasing the intake of a diversified diet and available foods for better MDD in the case of the Ghana study.

Even though FV and Animal Source Food (ASF) are known sources of vitamin A, protein and zinc, the most common foods used to feed infants are cereal-based gruels produced from traditionally refined cereal grains or tubers with minimal intake of FV, and ASF in developing countries^(27–29)^. The present study similarly found that the majority of children received primarily cereal (92⋅5 %), legumes (58 %) and milk (48 %) with a low intake of meat (14 %), vitamin A-containing FV (27⋅5 %) and other FV (20⋅8 %) which is almost similar to the review conducted in southern Ethiopia^(26)^. Regarding consumption of food items across agro-ecological districts, the low proportion of children who consumed vitamin A-containing FV in lowland compared with highland counterparts. This difference might be because the highland farmers were growing vitamin A-containing FV for consumption for household use in addition to cereal and legumes, while the lowland farmers were primarily growing cash crops such as coffee in addition to cereal production. Contrary, only a few children received fleshy meat with low prevalence in high and midland compared with lowland counterparts. A similar finding was reported by Roba KT^(15)^. A possible reason for the higher consumption of meat in the lowland could be that the cash crop is sold to the market and obtains adequate money to buy stuff that can cover the high cost of meat group. Low consumption of fleshy meat and vitamin A-containing FV might contribute to poor Dietary Diversity Score (DDS). In turn, it might compromise proper growth, development and immunity^(9,30)^. It showed that the more production diversity at the household level, the more children meet the requirements for MDD. Thus, agricultural production diversity is needed to improve the complementary foods diversity^(31,32)^.

In our study, the proportion of children (64⋅1 %) who met MMF was high. This finding is consistent with the review EDHS, Horo, Shasemene^(8,18,23)^. Moreover, the present study reported a higher rate of MMF in lowland areas (73⋅6 %). This finding is consistent with studies in Kenya^(6)^ and Ethiopia^(15)^. The reason for the higher prevalence of MMF in the lowland could be the agricultural fieldwork (coffee plantation) handled by the husband or employed person, thereby mothers have adequate time and stay at home to feed their children frequently. Whereas, in the high and midland, the majority of mothers participated in extraneous agricultural activities and they do not have time to feed their children per recommendation^(31)^.

The overall finding of MAD in this study is comparable with the results from developing countries: Ethiopia and Nigeria^(8,20,22,32)^. However, it was low compared with studies in Shashemene, Aceh and Congo^(23,25,33)^. This might be due to the difference in healthcare services, the residential area (urban *v*. rural) and the maternal illiteracy rate. The low MAD clearly shows that the majority of the children have failed to meet the required nutrient and energy density as a MAD is the composite indicator of MDS and MMF.

The present study confirmed that 42⋅7 % of the mothers were knowledgeable about complementary feeding practices. Finding from western Ethiopia (46⋅1 %)^(21)^ and Adea (51 %) were similar to the present study. But, it is lower than the study conducted in an urban area (68⋅4 %) of northwest Ethiopia^(34)^. The discrepancy could be because the mothers who lived in urban may have been accessing information through mass media compared with mothers who lived in the present study's rural area. Another explanation for the difference might be the present study used mean value as a cut-off to measure the mother's nutritional knowledge and classified it as poor and good. In northwest Ethiopia, studies employed principal component analysis to assess mothers’ knowledge through tertiles, then assigned as lowest, medium and highest. Thus, the classification in northwest Ethiopia might allocate much proportion of mothers as having good knowledge.

The multivariable analysis of this study indicated that maternal education, family size, residence, knowledge and decision on the use of money in the household were associated with OCFP after controlling for confounders.

Maternal education attainment of primary schooling was significantly associated with optimal child-feeding practice. This finding is consistent with the study from western Ethiopia and the review of mini EDHS 2019, Nigeria and Congo^(18,32,33)^. The possible reason could be mothers who attended primary and above school have more exposure to media for information on feeding practices. Hence, mothers could have better information to practice optimal complementary feeding.

Additionally, mothers who have good knowledge of complementary feeding practices were three times more likely to practice optimal complementary feeding compared with women who have poor knowledge. Studies conducted in Sidama, Adea and Pakistan supported this study^(20,21,35)^.

Another factor associated with optimal feeding practices was family size. Mothers with smaller family sizes (1–3 people) are more practised optimum complementary feeding with mothers having ≥4 persons in the household. The finding is consistent with a review of EDHS 2019 and Sidama in Ethiopia^(18,20)^. The possible explanation is that mothers with bigger family sizes might not offer a nutrient-dense diet and enough food for their children. Moreover, mothers may not have adequate time to prepare and feed their children according to the IYCF recommendation beyond the family dish.

To the best of our knowledge, no study assessed the association between OCFP of children aged 6–23 months of children among highland, lowland and midland agro-ecological zones in Ethiopia. However, the present study reported that those children from the highland areas were two times more likely to meet the optimal feeding practice compared with their midland area counterparts. This finding is similar to the study conducted in midland and lowland districts by Kedir *et al.* in Ethiopia^(15)^. Other studies from four Anglophone West African countries and Kenya showed the variation of complementary feeding practices across regions^(6,36)^. In Ethiopia, farmers predominantly grow cereals because the staple food is ‘Injera’ made up of cereal crops. Those farmers who lived in the highland had the experience of growing vegetables and fruit in addition to cereals and tubers. Therefore, a child from the highland areas had a higher probability of consuming four or more food groups compared with the lowland cereals-dependent areas. Thus, dietary diversity might contribute to higher optimal child-feeding practices for those children in the highland.

### Limitations

A single 24-h recall used in this study may not reflect the usual dietary practices of children. Moreover, the study did not gather data across all seasons and may not consider what is going on in different seasons, which should be considered in future studies.

## Conclusion

The overall findings of this study showed a low prevalence of OCFP, particularly in the midland area. Maternal educational status, family size, agro-ecological residence and knowledge were significant predictors of OCFP. The findings imply the need for multi-sectoral collaboration to strengthen maternal education, family planning service and the promotion of diversified agricultural production to improve optimal complementary feeding practices. Further research on the association of seasonal variation and child feeding practice should be recommended.

## Supplementary material

For supplementary material accompanying this paper visit https://doi.org/10.1017/jns.2023.26. click here to view supplementary material
