# Supplementary material for: Optimal complementary feeding practices of children aged 6–23 months in three agro-ecological rural districts of Jimma zones of southwest Ethiopia
Source: J Nutr Sci. 2023 Mar 27;12:e40. doi: 10.1017/jns.2023.26 (PMC10052435; doi:10.1017/jns.2023.26)
Supplement: Supplementary file 1 [file S2048679023000265sup001.docx]

Dedo=18k

Vegetable producer

3 districts based on aggro-ecological character

Proportion to size allocation

38

38

26

29

38

43

26

42

62

31

39

Kersa=26k

Cereal

producer

Mana=16 k

Coffee producer

K1

290

K2

200

K3

316

K4

239

K5

218

K6

112

K7

276

K1150

K2

62

K3

237

K4

120

K5

100

K6

145

K1

112

K2

145

K3

99

K4147

K5

164

77

52

83

63

57

29

72

**Systematic sampling technique**

**Fig 1**. **Schematic representation of Sampling technique, Jimma , South**
